# Fabrication and Characterization of a Ruthenium Nitride Membrane for Electrochemical pH Sensors

**DOI:** 10.3390/s90402478

**Published:** 2009-04-09

**Authors:** Yi-Hung Liao, Jung-Chuan Chou

**Affiliations:** 1 Graduate School of Engineering Science and Technology, National Yunlin University of Science and Technology / 123, 3 sec. University, Douliou, Yunlin, Taiwan R.O.C.; E-Mail: liaoih@tit.edu.tw; 2 Department of Information Management, Transworld of Institute of Technology / 1221, Jen-Nang Rd., Chia-Tong Li, Douliou, Yunlin, Taiwan R.O.C.; 3 Graduate School of Electronic Engineering, National Yunlin University of Science and Technology/ 123, 3 sec. University, Douliou, Yunlin, Taiwan R.O.C.

**Keywords:** Ruthenium nitride, ion selective electrode, temperature coefficient, light influence, drift rate, hysteresis width

## Abstract

The pH sensing and nonideal characteristics of a ruthenium nitride (RuN) sensing membrane pH sensor were investigated. RuN thin films were deposited from a 99.9% ruthenium target on p-type silicon substrates using radio frequency (r.f.) sputtering with N_2_ gas. Subsequently, the nanometric structure and surface morphology of RuN thin films were determined. The sensitivity of the RuN sensing membrane pH sensor was 58.03 mV/pH, obtained from I_D_-V_G_ curves with a current-voltage (I–V) measurement system in standard buffer solutions from pH 1 to pH 13 at room temperature (25 °C). Moreover, the nonideal characteristics of the RuN sensing membrane, such as temperature coefficient, drift with light influence, drift rate and hysteresis width, etc. were also investigated. Finally, the sensing characteristics of the RuN membrane were compared with titanium nitride (TiN), aluminum nitride (AlN) and silicon nitride (Si_3_N_4_) membranes.

## 1. Introduction

In the past to recent years, the measurement of pH values has been a very important parameter in different application fields, for example: clinical diagnosis, wastewater and environment monitoring, etc. The ion sensitive field effect transistor (ISFET) was first presented by Bergveld in 1970 [1]. Many hydrogen ion sensing gate materials are used for ISFET, for instance, a-Si:H [2, 3], Si_3_N_4_ [4], Ta_2_O_5_ [4], Al_2_O_3_ [4], a-WO_3_ [5], SnO_2_ [6] and PMT [7]. These ion sensing thin films are prepared using different methods, such as sputtering [5], sol-gel [6, 7], thermal evaporation [8] and plasma enhanced chemical vapor deposition (PECVD) [9, 10]. Subsequently, Van der Spiegel *et al*. [11] published the first paper on the extended gate field effect transistor (EGFET) in 1983. The configuration of the EGFET was separated into two parts: the sensing membrane and a metal oxide semiconductor field effect transistor (MOSFET) structure. The sensing thin films of ISFET or EGFET were almost always metal oxides. In this study, we will introduce other sensing membranes, the metal nitrides. Many researchers have discussed nitride thin films, which include titanium nitride (TiN) [12–16], hafnium nitride (HfN) [14], aluminum nitride (AlN) [17–21], indium nitride (InN) [22], chromium nitride (CrN) [23], zirconium nitride (ZrN) [24], gallium nitride (GaN) [25], ruthenium nitride (RuN) [26, 27] and silicon nitride (Si_3_N_4_) [28, 29], etc. There are many literature reports [5, 10, 19, 26, 30–35] which discuss the temperature and light nonideal characteristics of ISFETs. Nitride membranes used for pH measurement have been reported previously [15–17, 19]. Lei *et al.* [15] adopted titanium nitride as a pH-sensitive material based on an extended gate ISFET. Chin *et al*. [16] also introduced a titanium nitride membrane used as a sensing material in pH sensors with EGFET. Chiang *et al.* [17, 19] reported an AlN/SiO_2_ gate pH ion sensitive field effect transistor used for hydrogen ion concentration measurement and investigated the drift, hysteresis and temperature effects of the aluminum nitride membrane. Chou *et al.* [36] reported a separative structure extended gate H^+^-ion sensitive field effect transistor (SEGFET) with tin oxide (SnO_2_) thin films made using sol-gel technology. The SEGFET structure was similar to the EGFET one, but the former is easier to fabricate and package, and has lower costs than ISFET structures. In this study, ruthenium nitride (RuN) thin films deposited on silicon substrates were prepared from a ruthenium target using the r.f. sputtering technique. The sensitivity of the RuN sensing membrane with the SEGFET was determined using a Keithley 236 current-voltage (I–V) measurement system. The drift with light influence, temperature coefficient, drifts rate and hysteresis width of the nonideal characteristics of RuN-based pH sensor were also investigated with a voltage-time (V-T) measurement system. In addition, the experimental results are compared with those of other nitride and oxide pH-sensing materials.

## 2. Experimental

### 2.1. Materials and regents

Silicon wafer was used as the substrate of the ruthenium nitride sensing membrane pH sensor device. The silicon substrate was p-type, (100)-oriented, provided by the National Nano Device Laboratories (NDL, Taiwan). The RuN sensing membrane was prepared using a sputtering system and deposited onto the silicon substrate maintained at 25 °C by radio frequency sputtering from a 2-inch-diameter, 1/4 inch-thickness, 99.99% purity ruthenium target. Before sputter deposition, the silicon substrate and ruthenium target were placed in the chamber, and then sputtering was carried out. Acetone and methanol solvents were purchased from ACROS Co. Ltd. (USA) and used for cleaning the silicon wafer. All regents were analytical grade and used without further purification.

### 2.2. Fabrication of RuN sensing membranes

The silicon substrates were cleaned ultrasonically in acetone and methanol alternately for 15 minutes, leached in deionized (D.I.) water, and then dried with N_2_ gas. In this work, a total operating sputtering pressure of 10 mtorr in Ar-gas-mixed N_2_ for 1 hour was used. The gas flow ratio of the Ar: N_2_ was 1:2 (15:30 in sccm). The radio frequency power was 100 W, at 13.56 MHz. After the RuN was sputtered, the silicon wafer substrate was cleaned with deionized water and cut about 0.5cm x 0.5cm size. A conducting wire was bonded to the substrate with silver glue and then baked at 130°C for 30 minutes. The substrate was simple to package with epoxy resin and a 2 mm x 2 mm sensing window was left to detect hydrogen ion concentrations. Ruthenium metal is a good conductive material, and according to [26], ruthenium nitride has low resistivity as a conductive material.

### 2.3. Morphology of ruthenium nitride analysis

To examine the surface morphology of ruthenium nitride, a scanning electron microscope (SEM) and a scanning probe microscope (SPM) were used. A Philips XL-40FEG field emission scanning electron microscope was used to reveal the cross-section and nanometric structures of the ruthenium nitride thin film and a tapping mode atomic force microscope (Digital Instrument NS3a controller with D3100 stage) was used to measure the surface morphology of ruthenium nitride thin film.

### 2.4. Measurement systems

**Figure 1.**
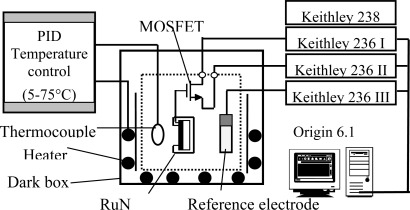
I–V measurement system used for the RuN pH-sensing membrane with separative extended gate field effect transistor.

A Keithley 236 semiconductor parameter analyzer was utilized to measure the current-voltage characteristics of the RuN thin film pH-sensitive SEGFET in pH = 1, 3, 5, 7, 9, 11 and 13 standard buffer solutions. The current voltage (I–V) measurement system is shown in Figure. 1. The gate of MOSFET is connected to the RuN pH sensor. The drain and source of MOSFET are connected to the current measuring unit of the Keithley 236. The RuN sensing membrane pH sensor device and an Ag/AgCl reference electrode were immersed into the different standard buffer solutions, respectively and placed inside a dark box to eliminate any light-induced effects. The commercial Ag/AgCl reference electrode provided a constant potential during the whole measurement process. The drain-source voltage (V_DS_) was maintained constant at 0.2 V and the V_G_ voltage was increased from 0 to 6 V while the drain-source current (I_DS_) was measured. The sensitivity of RuN sensing membrane pH sensor was obtained from the I_D_-V_G_ curves.

**Figure 2.**
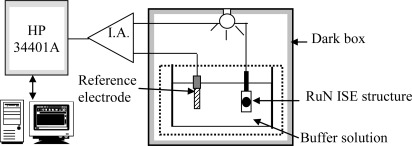
V-T measurement system used for the RuN sensing membrane pH sensor.

The voltage-time measurement system is shown in Figure 2. The V-T measurement system consisted of the PID temperature controller, an instrument amplifier (I.A.) as readout circuit and a HP 34401A multi-function digital meter voltage-time recorder. They were used to determine the drift and hysteresis behaviors of the RuN sensing membrane pH sensor. The sensor and reference electrode were immersed in the pH buffer solution for 12 hours, then the output voltage of the pH sensor was recorded; then the drift rate is the slope of the output voltage with respect to time, where the time is greater than five hours. The drift with light influence of the RuN sensing membrane test structures was investigated by illumination using a 110 V light bulb in a measurement chamber. The voltage variation was monitored with the light in the measurement environment on and off.

## 3. Results and Discussion

### 3.1. Materials analysis of ruthenium nitride thin films

**Figure 3.**
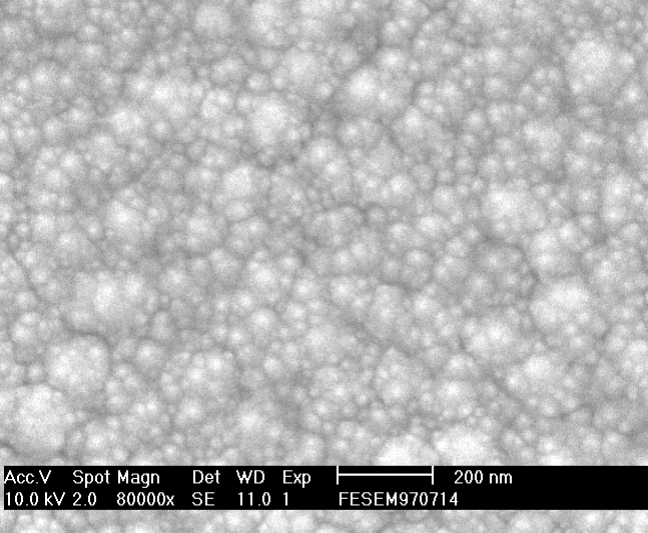
Nanometric structures of ruthenium nitride thin films by SEM measurement.

In this study, the membrane was the same as that used in our previous research [26], which showed that the thin film deposited using the sputtering system was ruthenium nitride. An Electron Spectroscopy for Chemical Analysis (ESCA) experiment was done on the RuN thin film and the results were consistent with the ESCA of ruthenium nitride thin film. The ESCA analysis exhibited XPS spectra which showed obvious Ru, N and O peaks. Moreover, the cross-section of ruthenium nitride measured from the scanning electron spectroscope (SEM) image in our previous research [38] showed a membrane thickness of ca. 574.2 nm. We used an atom force microscope (AFM) to investigate the morphological structure of the ruthenium nitride thin film. A field emission scanning electron spectroscope (FE-SEM) was used to investigate the nanometric structure of ruthenium nitride thin film. Figure 3 shows the typical SEM image pattern of the ruthenium nitride thin film deposited on the silicon substrate. The RuN surface in the 80,000 times SEM image shows surface compactness and some clustered nano particles. Scanning probe microscope experiments in tapping mode of operation was employed in order to determine the morphological structure of ruthenium nitride thin films.

**Figure 4.**
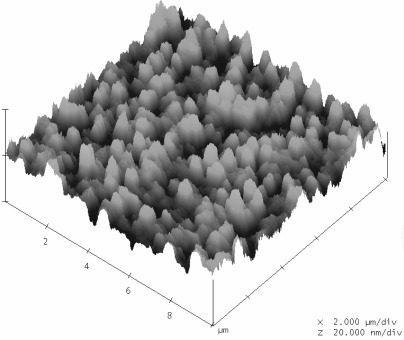
Surface morphology of ruthenium nitride thin films by AFM measurement

Figure 4 shows the morphology of the silicon wafer after the deposition of ruthenium nitride using the sputtering system. It displays the atomic force microscope image of the ruthenium nitride thin film, and the profile shows that the measured average (Ra) and root mean square (Rrms) of the roughness analysis of the ruthenium nitride particles are 2.42 nm and 3.12 nm, respectively. The nano scale of the surface can increase the reaction area with hydrogen ion analyte solution.

### 3.2. Characteristics of ruthenium nitride membrane pH sensor

#### 3.2.1. I–V characteristic and pH sensitivity

**Figure 5.**
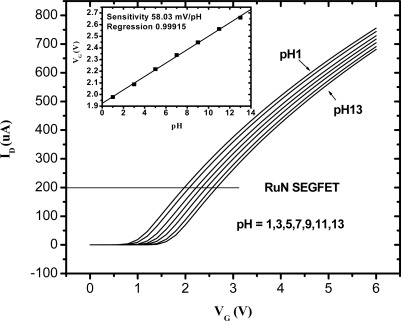
I–V and sensitivity curves of ruthenium nitride thin film pH sensor.

Figure 5 shows the I_D_-V_G_ characteristic curves of the RuN sensing membrane pH sensor in the pH range from pH 1 to pH 13 at room temperature (25 °C). According to [28], as the pH value increases, the surface potential of the ion-sensing membrane decreases. Therefore, the I_D_-V_G_ curve was shifted positively as the pH value increased. The inset of Figure 5 was obtained from the I-V curves of Figure 5. In Figure 1, the MOSFET was operated at a drain current of 200 μA, and the V_G_ voltages were between 1.975V and 2.656V in standard buffer solutions from pH 1 to pH 13. The V_G_ versus pH curves were obtained, and the sensitivity was defined as ΔV_G_/ΔpH and was shown in the inset of Figure 5. Therefore, we can obtain the sensitivity of RuN sensing membrane is 58.03 mV/pH in the buffer solutions from pH 1 to pH 13. Chin *et al.* [16] presented a titanium nitride membrane EGFET pH sensor and its sensitivity was 57.27 mV/pH in buffer solutions between pH 2 and pH 10. Chiang *et al*. [17] reported that the sensitivities of AlN pH electrodes were 48–57.25 mV/pH in the buffer solutions range of pH = 1–11. We can deduce that the sensitivity and pH measurement range of the RuN membrane pH sensor is superior to the AlN, TiN and Si_3_N_4_ membranes pH sensors. Comparisons of the sensitivity and pH range with the other nitrides and different thin films are listed in Table 1.

### 3.3. Nonideal characteristics of ruthenium nitride membrane pH sensor

The electrochemical pH sensors, such as ion selective electrode (ISE), EGFET and ISFET have exhibited some primary nonideal characteristics. The drift with light influence, temperature coefficient, drift rate and hysteresis width of ruthenium nitride membrane pH sensor were studied.

#### 3.3.1 Temperature coefficient

The response of an ideal pH electrode is defined by the Nernst equation as follows:

(1) $$E=E_{0}-2.3\frac{\mathrm{RT}}{\mathrm{nF}}\text{log}\;a_{H}^{+}$$

where:

E is total potential (in mV) developed between the sensing and reference electrode;
E_0_ is standard potential of the electrode at $a_{H}^{+}$ = 1 mol/L;
R is Gas constant;
T is temperature;
n is valency of ion;
F is Faraday constant;
$a_{H}^{+}$ is activity of the hydrogen ion in solution.

The term 2.3RT/nF is referred to as the Nernst slope. Table 2 shows the changes in Nernstian slope for the RuN electrode at increasing temperatures. The I–V characteristics measurements of the RuN pH sensor were carried out for pH 1∼pH 13 buffer solutions at 5 °C, 15 °C, 25 °C, 35 °C, 45 °C and 55 °C, respectively. The sensitivity value increased with increased operation temperature. From the data of Table 2, the temperature coefficient of sensitivity of the RuN pH sensor is 0.168 mV/pH°C. To investigate the temperature coefficient of RuN sensing membrane pH sensor the temperature compensation data was used. Furthermore, the temperature coefficients in pH electrochemical sensors with various sensing materials, such as TiN, and Si_3_N_4_ have been also reported [16, 17, 35]. Chin *et al*. [16] described that the temperature coefficient of sensitivity of TiN pH sensor is 0.180 mV/pH°C. Chiang *et al*. [17] reported that the temperature coefficient of sensitivity of the AlN pH sensor is 0.130 mV/pH°C. Chou *et al*. [35] presented that the temperature coefficient of sensitivity of Si_3_N_4_ pH sensor is 0.150 mV/pH°C.

#### 3.3.2. Drift with light influence

**Figure 6.**
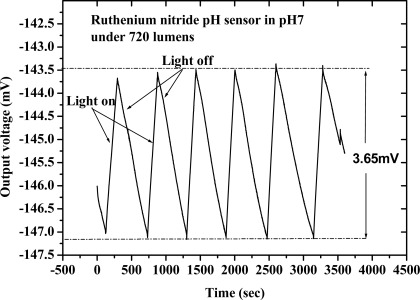
Drift with light influence of RuN thin film pH sensor.

Voorthuyzen and Bergveld [33] were the first to investigate the slow response of the Ta_2_O_5_ gate ISFET after illumination. In this study, we investigated the drift with light influence characteristics of the RuN thin film pH sensor in the dark box and under constant light exposure, respectively. Our experiment was focused on the output voltage variation of the pH sensor after switching the light on and off. The experimental results are shown in Figure 6. When the light was switched on and off the output voltage of the RuN pH sensor varied with time. Then the 3.65 mV variation amount of response voltage was obtained with light on and light off and this voltage will make a 0.063 pH (3.65 mV/58.03 (mV/pH)^−1^) variation.

#### 3.3.3 Drift effect

**Figure 7.**
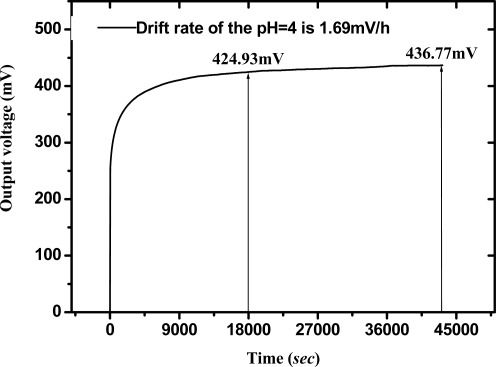
Drift rate of RuN pH sensor in pH 4 buffer solution for measuring 12 hours.

The drift phenomenon in pH electrochemical sensors with various sensing materials, such as a-Si:H, Si_3_N_4_, Al_2_O_3_, Ta_2_O_5_, AlN and SnO_2_ has been discussed [3, 4, 6, 8, 10, 17]. According to Zhong *et al.* [37] the surface voltage response reaches a steady state after five hours with no pH variation. Therefore, we choose the measured data as representing a long term drift rate during 5–12 hours. The drift phenomenon shows that the output voltage of the RuN sensor device varies slowly with time. Drift behavior existed in the whole measurement process and could be characterized as a function of pH value. The V-T measurement system was used to measure the drift rate of the RuN sensing membrane pH sensor in pH1, 4, 7, 10, 13 standard buffer solutions. Figure 7 shows the drift rate of RuN pH sensor in pH4 buffer solution after measuring for 12 hours. The calculation of drift rate is 1.69 mV/h, which is the voltage difference from 18,000 sec (5 hours) to 43,200 sec (12 hours), divided by the time duration (7 hours). According to the experimental results, we can obtain the drift rate with percent error of the RuN sensing membrane pH sensor, which are shown in Table 3. It is obvious that the higher the hydrogen ion concentration, the lower the drift rate was. The cause is that at lower H^+^ concentrations, the higher pH value, and the ion size of H^+^ is smaller than OH^−^. Therefore, the drift speed of H^+^ is quicker than for OH^−^. The drift rates were compared with other nitrides and different thin films as shown in Table 1.

#### 3.3.4. Hysteresis effect

The RuN pH sensor was measured many times in the same pH buffer solution to see the difference of output voltages. This phenomenon we called hysteresis or memory effect. According to Bousse *et al*. [32] the hysteresis of pH-ISFETs could be regarded as a delay of the pH response. The hysteresis only occurs at the buffer solution and insulator interface, and is related with the buffer solution composition. The V-T measurement system was used to measure the hysteresis width of the RuN sensing membrane pH sensor in different pH buffer solutions with a pH loop cycle and 10 minutes loop time. According to the experimental results, the hysteresis widths of the RuN sensing membrane pH sensor are 2.7 mV and 9.1 mV in pH 7-4-7-10-7 and pH 7-10-7-4-7, respectively. We also compared the hysteresis width with the different thin films, as shown in Table 4.

## 4. Conclusions

In this study, we use the sputtering method to prepare a RuN sensing membrane as an electrochemical pH sensor. The sensing characteristics of the ruthenium nitride membrane were stable in pH buffer solutions between pH 1 and pH 13. From the experimental results, the sensitivity of the RuN sensing membrane pH sensor is 58.03 mV/pH. Therefore, we conclude that the RuN membrane pH sensor is better than TiN, AlN and Si_3_N_4_ membrane ones with regards to pH range and sensitivity, respectively. The temperature coefficient of the RuN pH sensor is 0.168 mV/pH°C from 5 °C to 55 °C. Light exposure resulted in a 0.063pH variation for the RuN pH sensor. The drift rates of the RuN sensing membrane pH sensor are 1.69 mV/h, 2.15 mV/h and 2.51 mV/h at pH 4, pH 7 and pH 10, respectively and the hysteresis widths of the pH 7-4-7-10-7 and pH 7-10-7-4-7 cycles are 2.7 mV and 9.1 mV, respectively.

## Figures and Tables

**Table 1.** Comparison of sensitivity, pH range and drift rate with other nitrides and different thin films.

**Thin film**	**Preparation method**	**Drift rate (mV/h)**	**Device structure**	**pH range**	**Sensitivity (mV/pH)**	**Ref.**
RuN	Sputtering	2.15	SEGFET	pH1-13	58.03	This study
Si_3_N_4_	PECVD	1.0	ISFET	pH1-13	46–56	[4]
AlN	Sputtering	2.43	ISFET	pH1-11	48–57.25	[17]
TiN	Sputtering	-	EGFET	pH2-10	57.27	[16]
a-WO_3_	Sputtering	15.7	ISFET	pH1-7	45–56	[5]
SnO_2_	Sputtering	9.1	EGFET	pH2-12	58	[10]
SnO_2_	Thermal Evaporation	28	ISFET	pH2-12	58	[8]
SnO_2_	Sol-gel	6.73	ISFET	pH1-9	57.36	[6]
PMT	Sol-gel	0.4	ISFET	pH2-12	58–59	[7]
a-Si:H	PE-LPCVD	6.53	ISFET	pH1-7	52.3	[3]
Ta_2_O_5_	PECVD	0.5	ISFET	pH2-12	56–57	[4]
Al_2_O_3_	PECVD	0.1–0.2	ISFET	pH1-13	53–57	[4]
TiO_2_	MOCVD	11.9	TiO_2_/SiO_2_/Si	pH3-11	57.2	[34]

**Table 2.** Sensitivity of RuN thin film pH sensor at various temperatures.

**Temperature (°C)**	**5**	**15**	**25**	**35**	**45**	**55**
Sensitivity (mV/pH)	55.51	57.47	58.03	60.52	62.35	64.04

**Table 3.** Drift rates of RuN thin film for the pH sensor.

**pH**	**pH 1**	**pH 4**	**pH 7**	**pH 10**	**pH 13**
Drift rate (mV/h)	1.09±0.5%	1.69 ± 0.8%	2.15± 1.4%	2.51± 3.9%	3.17± 4.2%

**Table 4.** Comparison of hysteresis widths for the different thin films.

**Thin film**	**Hysteresis width (mV)**	**Loop path**	**Loop time (min)**	**Ref.**
RuN	2.7	pH 7-4-7-10-7	10	In this study
	9.1	pH 7-10-7-4-7	10	
TiN	0.5	pH 7-4-7-10-7	10	[16]
AlN	1.0	pH 7-3-7-11-7	16	[17]
Si_3_N_4_	2.0	pH 7-3-7-11-7	1024	[39]
a-Si:H	17.9	pH 3-1-3-5-3	-	[2]
	1.5	pH 3-5-3-1-3	-	
a-WO_3_	1.5	pH 3-5-3-1-3	-	[5]
	26.0	pH 4-7-4-1-4	10	
SnO_2_	1.3	pH 4-1-4-7-4	13	[6]
	3.74	pH 5-1-5-9-5	17	
PMT	1.0	pH 7-4-7-10-7	25	[7]
SnO_2_	9.8	PH 7-4-7-10-7	10	[10]
